# Cryoablation for the treatment of chronic rhinitis: a systematic review

**DOI:** 10.1186/s40463-023-00645-6

**Published:** 2023-04-29

**Authors:** Veeral Desai, Gianluca Sampieri, Amirpouyan Namavarian, John M. Lee

**Affiliations:** 1 Faculty of Medicine, Queen’s University, Kingston, ON Canada; 2 Department of Otolaryngology – Head and Neck Surgery, University of Toronto, Toronto, ON Canada; 3 Division of Rhinology, Department of Otolaryngology - Head and Neck Surgery, St. Michael’s Hospital, Unity Health Toronto, Toronto, ON Canada

**Keywords:** Rhinitis, Endoscopic sinus surgery, Chronic disease

## Abstract

**Background:**

ClariFix is a novel intranasal cryotherapy device developed for clinic-based cryosurgical ablation of the posterior nasal nerves region. As a relatively new technology, there is a paucity of studies within the literature assessing the efficacy and safety profile of ClariFix for chronic rhinitis.

**Methods:**

A systematic review was completed in accordance with PRISMA guidelines. Databases searched included: Ovid Medline, Ovid EMBASE, Pubmed, Cochrane and Web of Science. Inclusion criteria consisted of studies investigating the use of ClariFix in chronic rhinitis (i.e., allergic and non-allergic rhinitis) in patients of all ages.

**Results:**

The initial search identified 1110 studies. Final analysis consisted of 8 articles, evaluating a total of 472 patients. The data showed a significant reduction in scores post-treatment across all studies based on validated outcome measures. In all studies, at all time intervals, there was a significant improvement in outcome scores from baseline. Minor adverse effects included post-procedural pain and discomfort, headache and palate numbness. No major adverse events were identified.

**Conclusion:**

ClariFix is a novel intranasal cryotherapy device that was introduced in Canada in 2021. This is the first systematic review evaluating its efficacy and safety profile. Across all studies, there was a significant reduction in validated outcome scores at multiple time intervals. Further, the treatment is safe with only minor adverse effects reported by patients. Overall, the consensus from this study highlights an apparent benefit in using this intervention for chronic rhinitis that is refractory to medical management.

## Introduction

Chronic rhinitis is a common disease with an estimated 320 million people affected worldwide [1]. A study that surveyed the Canadian population reported that approximately 15% had chronic or recurrent rhinitis or sinusitis [2]. Pharmacologic interventions such as intranasal anticholinergics, antihistamines, and decongestants are usually the first line of therapy for rhinitis; however, these first-line therapies fail to control symptoms due to lack of efficacy or intolerance to treatment in 10–22% of patients [3, 4]. For these patients that have rhinitis refractory to medical treatment, surgical interventions may be indicated [3, 4]. Historically, vidian neurectomy and posterior nasal nerve (PNN) sectioning were used to disrupt preganglionic parasympathetic innervation to the nasal mucosa to decrease secretions and symptoms in patients [3, 5]. The risk of serious complications and the need for general anesthesia have limited the broad acceptance of both vidian neurectomy and PNN resection despite their effectiveness [6].

**Fig. 1:**
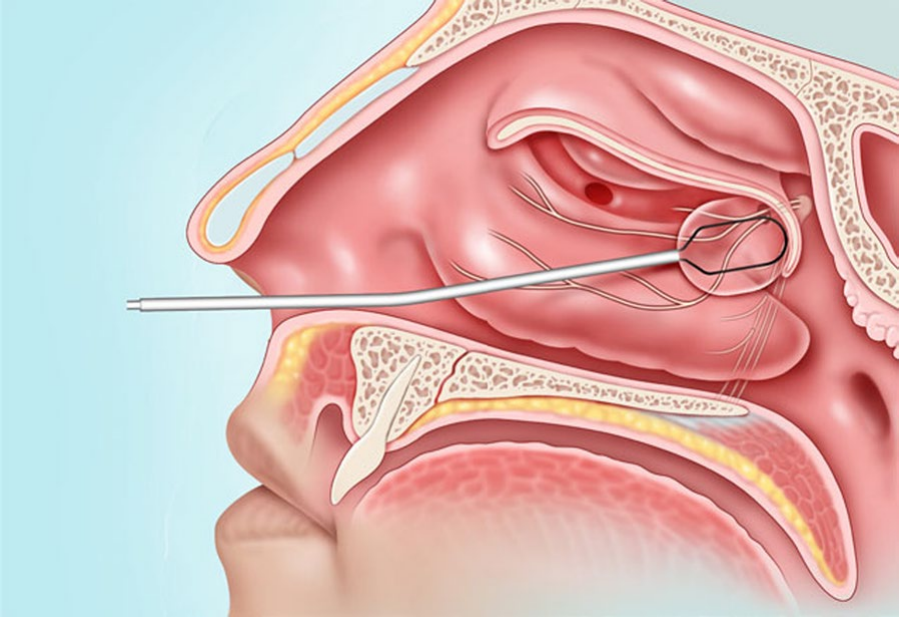
Intranasal device position for ClariFix™ application

Cryotherapy is a surgical technique that offers the advantage of ablating soft tissue and nerve with predictable depth of penetration, preserving arterial vascular supply to the region and minimizes the risk of necrosis [7]. A novel transnasal cryotherapy device (ClariFix™) has been developed for clinic-based cryosurgical ablation of the PNN region, requiring only local anesthesia. This device was approved for use in the United States in June 2016 and Canada in June 2021 as a less invasive surgical option for rhinitis refractory to medical management [8, 9]. The device is a hand-held, endoscopically placed cryoprobe through which nitrous oxide cryogen is delivered at the tip in a closed system to ablate the nerves [8]. An image of internasal device position is visible in Fig. 1, courtesy of Stryker [8]. A qualitative systematic review in 2018 commented that although cryotherapy appeared safe and efficacious, heterogeneous and low-quality evidence made strong, evidence-based assessments difficult [6]. Since then, a number of prospective studies have been published assessing the efficacy and potential side effects of ClariFix™. These studies have shown significant reductions in nasal symptom scores and quality of life through validated metrics [10, 11]. Additionally, a database analysis published by Singh et al. in 2021, highlighted minor adverse events such as epistaxis and nasal swelling in ClariFix™ use [12]. The aim of this study is to assess the efficacy and adverse outcomes of ClariFix™ use by pooling existing data, thereby increasing the sample size.

## Methods

### Search strategy

**Fig. 2:**
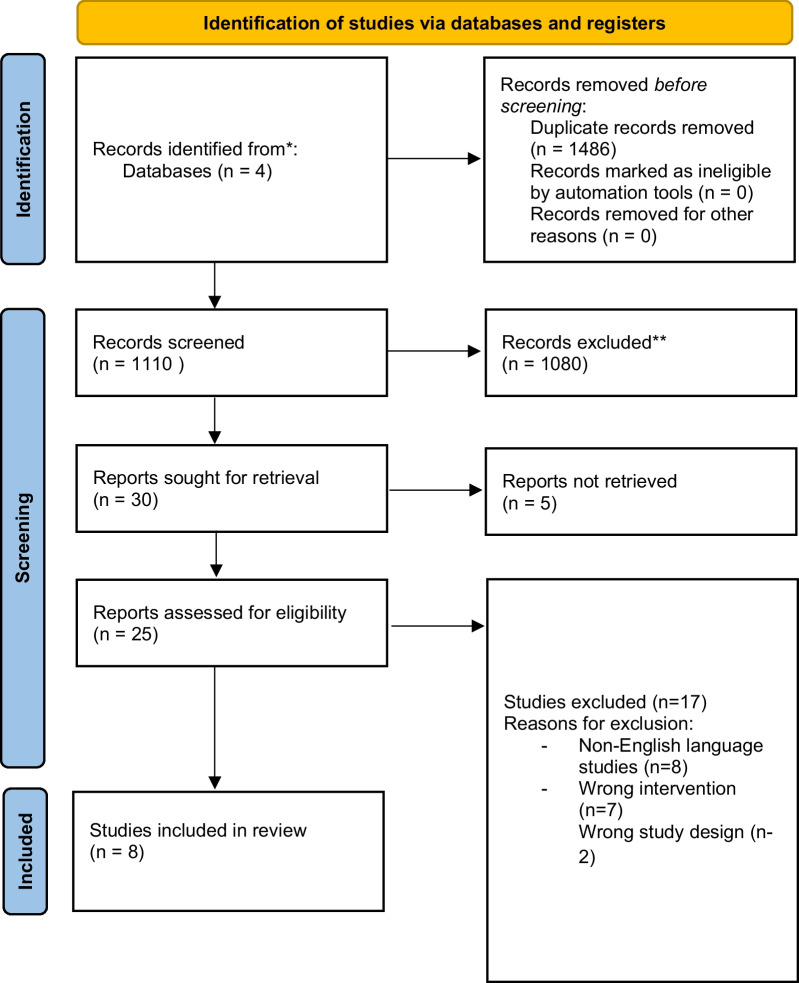
Preferred Reporting Items for Systematic Reviews and Meta-Analyses flowchart

This systematic review was completed in accordance with the Preferred Reporting Items for Systematic Reviews and Meta-Analyses (PRISMA) guidelines (Fig. 2). The database searches were performed by two reviewers (V.D. / G.S.). Databases searched included: Ovid Medline, Ovid EMBASE, Pubmed, Cochrane and Web of Science. The search was completed from database inception (1946) to November 23, 2021. Keywords and Medical subject headings (MeSH) that were searched included: cryoablation, cryosurgery, cryotherapy, cryosurgical ablation, posterior nasal nerve cryoablation, posterior nasal nerve, chronic rhinitis, rhinitis, allergic rhinitis, non-allergic rhinitis, vasomotor rhinitis, rhinorrhea and congestion.

### Inclusion and exclusion criteria

Inclusion criteria consisted of studies investigating the use of ClariFix under local anesthesia in chronic rhinitis refractory to medical management (i.e., allergic and non-allergic subtypes) in patients of all ages. We originally defined chronic rhinitis from literature as inflammation of the nasal lining for > 4 weeks that can be further sub-classified into allergic and non-allergic subtypes differentiated based on percutaneous skin test and the allergen-specific immunoglobulin E (IgE) antibody test [13]. Of note, there was heterogeneity between the various included studies on the definition of chronic rhinitis. It is important to note that some studies such as Chang et al. established different criteria (Table 1). Randomized controlled trials, prospective or retrospective observational studies, cross-sectional and case–control trials were included. Studies that did not utilize ClariFix™ were excluded. Papers published in a non-English language or a non-peer reviewed journal were excluded. Abstracts, conference posters, reviews, letters to editors, and editorials were excluded.

**Table 1:** Study characteristics

Author and reference	Study design	Sample size	Key chronic rhinitis criteria	Sex (M/F)	Mean age (range), years	Outcome measure	Industry sponsor
Del Signore et al. [14]	RCT	133	Symptoms have been chronic for 6 months or longer, minimum TNSS score of 4	CT: 23/45 Sham: 33/32	CT: 52.3 ± 15.8 Sham: 58.3 ± 16.4	TNSS RQLQ NOSE	Stryker Corporation
Chang et al. [11]	Prospective single-arm trial	98	Minimum rTNSS score of 4, with a minimum score of 2 for rhinorrhea and 1 for nasal congestion	35/63	58.6 ± 16.2	rTNSS RQLQ	Stryker Corporation
Gerka Stuyt et al. [10]	Prospective single-arm trial	24	Not explicitly defined	12/12	60.04 ± 16.7	TNSS	None Declared
Hwang et al. [15]	Prospective single-arm trial	27	Minimum rTNSS rhinorrhea and/or congestion subscore of 2	10/17	53.3 ± 3.3	TNSS	Arrinex, Inc. (Stryker Corporation)
Yen et al. [16]	Prospective single-arm trial	30	Severe rhinorrhea and mild to severe nasal congestion lasting at least 3 months	14/16	60.0 ± 15.8	TNSS NOSE SNOT-22 Nasal Symptom VAS Mini RQLQ	Stryker Corporation
Ow et al. [8]	Prospective, multicenter, interventional, single-arm	91	Chronic rhinitis for 6 months or longer, minimum TNSS score of four, with a minimum score of two for rhinorrhea, one for congestion	36/64	58.8 ± 16.2	rTNSS RQLQ	Arrinex, Inc. (Stryker Corporation)
Virani et al. [17]	Retrospective cohort study	14	Patients who has persistent allergic or non-allergic rhinitis	9/5	59.1	rTNSS RQLQ	None Declared
Yoo et al. [18]	Multi-institutional retrospective case–control study	55	Not explicitly defined	(25/30)	55.3 ± 17.2	RNS	None Declared

### Data extraction and analysis

The search titles and abstracts were independently screened by two reviewers (V.D./G.S.) based on the aforementioned inclusion and exclusion criteria. Next, full manuscripts were retrieved and independently reviewed by the same two reviewers. Any disagreements in article selection between the two reviewers were resolved by consensus. If a disagreement persisted, a third reviewer was consulted (A.N.). All title, abstract, and full text screening was completed using Covidence (version 1501). The references of relevant articles were searched to identify potential studies that discussed the use of ClariFix in chronic rhinitis. A standard data extraction template categorized in a shared document was used to collect the data. Extracted data included study design, study population demographics (size, sex, age, treated condition), follow-up period, outcome metric (i.e., Total Nasal Symptom Score, Rhinoconjunctivitis Quality of Life Questionnaire) and adverse events. Due to significant heterogeneity in follow-up time and outcome metrics, only descriptive statistics could be used in this study.

### Risk of bias assessment

Studies were evaluated for risk of bias using the Cochrane Collaboration Tool for Risk of Bias Assessment for RCTs and the Newcastle–Ottawa Quality Assessment Scale for cohort studies [19, 20] (Tables 3, 4). Two authors assessed the risk of bias according to this tool. All disagreements were resolved by way of discussion. These tools were used to evaluate the risk of studies underestimating or overestimating the effect of ClariFix use for chronic rhinitis.

## Results

The initial search identified 1110 studies. After title and abstract screening, 30 articles met the criteria for full-text review. Eight studies were excluded due to foreign language, 5 were inaccessible, and the remainder did not fulfill inclusion criteria. No studies assessed through a bibliographic review of key references met the inclusion criteria. Final inclusion consisted of 8 articles that specifically evaluated the use of ClariFix in chronic rhinitis (Fig. 1). Of the 8 studies included, one was an RCT and 7 were single-arm cohort studies (5 prospective and 2 retrospective). A total of 472 patients were evaluated across all studies. The results are summarized in Table 1. Of the studies, 6 of them had sub-cohorts of chronic allergic, nonallergic rhinitis, and mixed rhinitis.

### Study outcomes

The efficacy of ClariFix for chronic rhinitis was measured via a number of validated symptom scoring questionnaires used to quantify the degree of symptom improvement. The most common scales used within this study were the Total Nasal Symptom Score (TNSS) and rTNSS scales. The Total Nasal Symptom Score (TNSS; possible score of 0–12) is the sum of 4 individual participant-assessed symptom scores for rhinorrhea, nasal congestion, nasal itching, and sneezing, each evaluated using a scale of 0 = None, 1 = Mild, 2 = Moderate, or 3 = Severe. The rTNSS was performed in the morning (AM rTNSS) and evening (PM rTNSS) and assessed the participant's symptoms over the preceding 12 h. The daily rTNSS is the average of the AM rTNSS and PM rTNSS assessments. Mean changes from baseline over the entire treatment period were calculated as treatment period rTNSS minus baseline rTNSS. This scale was used in 7 of the 8 included studies and the minimal clinically important difference for rTNSS has been defined as a 30% reduction in baseline score [14].

### rTNSS/TNSS

**Fig. 3:**
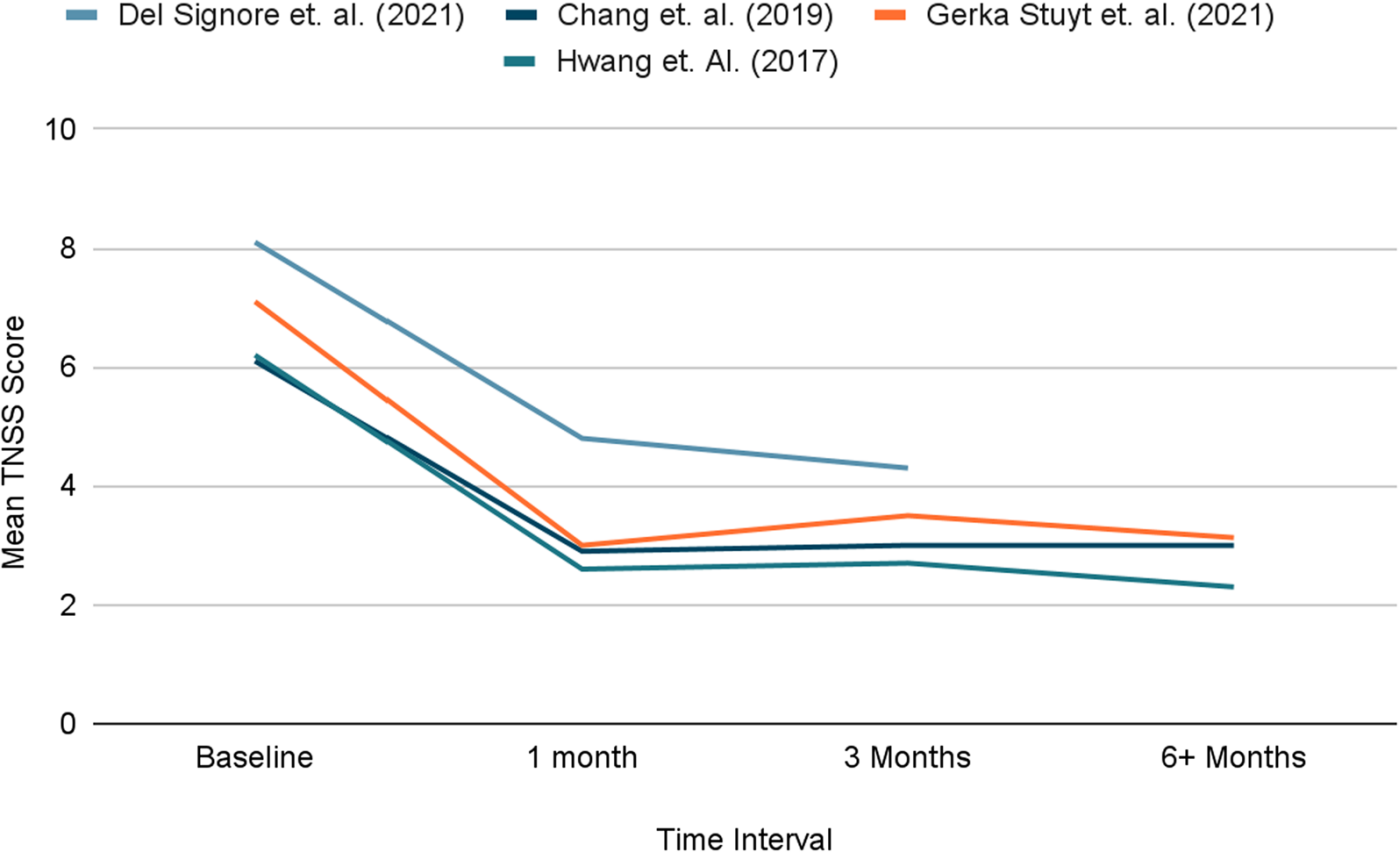
Mean TNSS scores at various time intervals

Of the seven studies that utilized the TNSS/rTNSS score, all showed significant improvement in symptoms. The average baseline score for the 7 studies that utilized the TNSS/rTNSS scores was 7.12. Four of the seven studies had an end-point assessing symptom improvement after one month and three months. Del Signore et al. conducted a randomized control trial of 133 patients that saw baseline mean TNSS scores fall from 8.1 ± 1.7 to 4.8 ± 2.3 and 4.3 ± 2.4 at 1- and 3-months post-procedure, respectively [14]. This significant reduction was also found in Chang et al., Gerka et al., and Hwang et al. where baseline mean TNSS scores fell from 6.1 ± 1.9, 7.1 ± 3.1, and 6.2 ± 0.5 to 2.9 ± 1.9, 3.0 ± 2.0 and 2.6 ± 0.3, at the 1-month time intervals respectively. For all three studies, this statistical improvement and clinically important difference was maintained at the 3-month time interval and beyond [10, 11, 15] (Fig. 3).

Follow-up length ranged from post-operatively to 24 months following cryoablation. In all studies, at all time points, there was a significant improvement in TNSS/rTNSS scores from baseline at respective time intervals.

### Other outcome measures

The one study that did not utilize TNSS/rTNSS scores assessed patients using the validated Sino-nasal Outcome Test (SNOT-22) by Yoo et al. [18] and Kennedy et al. [21]. This retrospective case–control study evaluated whether disease features and ipratropium nasal spray response predicted rhinorrhea response after PNN cryoablation. This study had patient-reported outcomes determined using the Runny Nose Score (RNS) from the 22-item Sino-Nasal Outcomes Test (SNOT22). The study found that rhinorrhea response to ipratropium was predictive of rhinorrhea improvement after PNN cryoablation and had a mean pre-procedural SNOT-22 RNS of 4.2 ± 1.0 and found that after cryoablation there was a > 1-point decrease in RNS in 71% of patients. Post-procedure SNOT-22 RNS scores were significantly decreased at the first and second follow-ups, although follow-up intervals were not specified [18]. Additionally, other validated scales were used in this study such as the nasal symptoms visual analog scale (VAS), Rhinoconjunctivitis Quality of Life Questionnaire (RQLQ), and physician perception of improvement using the Clinical Global Impression–Improvement (CGI–I). These measures also showed significant improvements following ClariFix intervention [8, 16]. Overall, there was significant variation in the outcome measure used across the studies. 7 studies used the TNSS or rTNSS scales, 5 studies used the RQLQ or mini-RQLQ scores, 2 studies used the NOSE score and 1 study used the SNOT-22, Nasal Symptom VAS and RNS scales (Table 1).

### Side effects/safety profile

No serious adverse events were reported in any of the studies. The most common adverse effect reported was post-procedural pain or discomfort. The incidence of this was very common, ranging up to 74% in some patients according to Hwang et al. [15]. However, this pain was reported as mild and did not persist. Other common complications included headache and palate numbness. A summary of the incidence of adverse effects can be seen in Table 2.

**Table 2:** Safety profile of ClariFix™

Adverse effect	Complication rate among all studies
Post-procedure pain	13.5% (64/472)
Headache	4.23% (20/472)
Numbness	2.96% (14/472)
Nasal congestion/sinusitis	1.48% (7/472)
Bleeding	1.06% (5/472)
Watery eyes	0.64 (3/472)

### Risk of bias

As mentioned above, studies were evaluated for risk of bias using the Cochrane Collaboration Tool for Risk of Bias Assessment for RCTs and the Newcastle–Ottawa Quality Assessment Scale for cohort studies. The results can be seen in Tables 3 and 4. The risk of bias for the single RCT included ranged from “low” to “unclear,” with no domain classified as having a “high” risk of bias. After converting the Newcastle–Ottawa Scale to the Agency for Healthcare Research and Quality standards for the included cohort studies, 6 studies (86%, 6 of 7) and 1 study (14%, 1 of 7) were rated as good and fair, respectively.

**Table 3:** Collaboration Tool for Risk of Bias Assessment for RCTs

Study (year)	Sequence generation	Allocation concealment	Blinding of participants and personnel	Blinding of outcomes	Incomplete data outcome	Selective reporting bias	Free of other sources of bias
Del Signore [14]	Unclear	Unclear	Unclear	Low	Low	Low	Low

**Table 4:** Newcastle–Ottawa Quality Assessment Scale for cohort studies

Quality Assessment Criteria	Acceptable	Chang et al. [11]	Gerka Stuyt et al. [10]	Hwang et al. [15]	Yen et al. [16]	Ow et al. [8]	Virani et al. [17]	Yoo et al. [18]
Selection								
Representativeness of exposed cohort	Individuals with chronic rhinitis undergoing ablation	*	*	*	*	*	*	*
Selection of the non-exposed cohort	Drawn from same community as exposed cohort							
Ascertainment of exposure?	Secured records, structured interview	*	*	*	*	*	*	
Demonstration that outcome of interest was not present at start of study?	Measurement of pre-treatment and post-treatment symptom scores	*	*	*	*	*	*	*
Comparability								
Study controls for age, sex	Yes	*	*	*	*	*	*	*
Study controls for any additional factor	Concurrent medication use	*			*	*		
Outcome								
Assessment of outcome	Standardized and validated assessment tool (rTNSS, TNSS, SNOT22)	*	*	*	*	*	*	*
Was follow-up long enough for outcome to occur?	Yes, treatment duration > 4 weeks	*	*	*	*	*	*	*
Adequacy of follow-up of cohorts?	All subjects accounted for or small number (< 5%) lost to follow-up or description provided of those lost	*	*	*	*	*		*
**Overall Quality Score (Maximum = 9)**		**8**	**7**	**7**	**8**	**8**	**6**	**6**
		Good	Good	Good	Good	Good	Good	Fair

Asterisk implies the feature was present in the respective studies. Bold row highlights the overall quality score

## Discussion

The aim of this study was to assess the efficacy and safety profile of ClariFix™ by pooling existing studies. The data from included studies indicated a significant reduction in scores post-treatment across all studies based on validated outcome measures. All studies demonstrated a significant improvement in outcome scores from baseline at every time interval. Minor adverse effects included post-procedural pain and discomfort, headache and palate numbness. No major adverse events were identified.

ClariFix™ is a relatively new intervention in the management of chronic rhinitis that has been introduced for clinical practice in Canada since 2021 [9]. This device can be used as an in-clinic procedure to allow for cryotherapy ablation of the posterior nasal nerve. The device uses nitrous oxide cryogen to ablate the nerves via a small endoscopically placed cryoprobe at the posterior nasal nerves [8]. This systematic review has amalgamated the results of 8 studies assessing the efficacy of the ClariFix™ cryotherapy intervention in treating chronic rhinitis. Risk of bias analysis revealed an overall low risk of bias using the Cochrane Collaboration Tool for Risk of Bias Assessment for RCTs and the Newcastle–Ottawa Quality Assessment Scale for cohort studies. Overall, the consensus from this study highlights a significant benefit in using this intervention for chronic rhinitis that is refractory to medical management. All studies, irrespective of outcome measure, showed a statistically significant improvement in symptoms or quality of life following ClariFix™ intervention. This was explicitly indicated by the studies that used the TNSS score where statistically significant improvement and clinically important differences from baseline were found at the 1- and 3-month time intervals across all studies. [10, 11, 14, 15]. For several of these studies, this improvement was followed and maintained at 6 months or beyond [10, 11, 15].

Cryotherapy has historically had a positive safety profile attributed to the limited depth of penetration, minimizing the potential impact on surrounding bone and cartilage. Preservation of connective tissues and the extracellular matrix not only provides a scaffold for tissue repair and optimal healing but also minimizes the potential of damage to larger blood vessels [22, 23]. Adverse effects are still present, the most common of which being post-procedural pain and discomfort, headache and palate numbness. No major adverse events were reported in any of the studies. This differs slightly from a database study that examined the adverse effects of ClariFix™. Singh et al. found the most common adverse events associated with ClariFix™ cryoablation include epistaxis and nasal swelling [12]. Notably, within that study, 5 of 9 epistaxis adverse events identified involved patients with a history of hypertension [12]. This is a significant confounding variable as there is evidence to suggest epistaxis is both more common and more severe in hypertensive patients [24]. As an aside, it should be noted that in the control group of the Del Signore et al. study, which was a sham procedure, participants reported adverse effects of post-procedure pain/discomfort (n = 1), vasovagal syncope (n = 1), and vomiting (n = 1) [14].

ClariFix™ cryotherapy presents a viable interventional option for chronic rhinitis that is refractory to medical management compared to other surgical options such as turbinate reduction and vidian neurectomy. The primary advantage is the clinic-based applicability of ClariFix™ and the lack of general anesthesia requirements [8, 25]. Additionally, there have been far lower rates of dry eye complications with cryotherapy, a common side effect of vidian neurectomy due to ablation of parasympathetic innervation to the lacrimal gland [3, 26]. Studies of vidian neurectomy have shown a similar reduction in symptom burden, with similar results based on the quality of life scores and reductions in nasal symptoms based on the visual analogue scale [27, 28]. However, there have been no studies directly comparing the efficacy of the different interventions [3, 25].

Peripheral neuroregeneration can occur at a rate of 1 to 6 inches per month, thus evaluating results beyond a year is important to determine the long-term efficacy of cryoablation treatment [29]. Three studies reported on longer-term outcomes, 12 months or longer, with statistically significant results. This highlighted the safety and durability of the ClariFix™ intervention [8, 10, 11].

### Limitations

This systematic review has several limitations. There is only a limited quantity of published studies on the use of ClariFix™ cryoablation for the treatment of chronic rhinitis. Additionally, of the 8 studies reviewed, only 1 of them was an RCT. The remaining seven were single-armed experimental studies with no comparator group, thus performing a meta-analysis of the data was not possible. Additionally, due to an inability to access the raw data from the included studies, a statistical analysis of the pooled means was not able to be performed. Another limitation of the study is highlighted by the variability of study reporting. Several different outcome measures were used across the various studies (Table 1). Additionally, one study opted to utilize the median values instead of means when reporting outcomes [16]. While studies published mean values and variances, data for individual patients was not available. An inability to access the individual participant scores at baseline and the various time intervals limited the ability to properly calculate pooled means and variance. Furthermore, five of the eight included studies were industry sponsored by Stryker corporation, the device manufacturer. This presents a potential conflict of interest, although the impact of this on the overall study findings are unclear.

Therefore, more independent, high-quality randomized controlled trials are required to perform a meta-analysis analyzing the effect of ClariFix™ on chronic rhinitis. Nevertheless, this systematic review is novel as it represents the only study that has amalgamated the current evidence on the efficacy and safety profile of ClariFix™ for chronic rhinitis and thus provides a preliminary review of the data to highlight the benefits and side effects of ClariFix™.

Additionally, our report does not delineate cryoablation between allergic vs non-allergic chronic rhinitis. A number of the individual studies did make reference to allergic vs non-allergic sub-cohorts, however due to the same structural limitations regarding heterogeneity in outcome measures and reported time intervals, the data was not aggregated and analyzed [11, 14, 15]. However, this represents a future direction for systematic review and meta-analysis once further comparator trials are published.

## Conclusion

ClariFix is a novel intranasal cryotherapy device that was introduced in Canada in 2021. This is the first systematic review evaluating its efficacy and safety profile. This systematic review demonstrates that ClariFix™ is safe and efficacious as a treatment modality for chronic rhinitis. Clinically meaningful reductions in nasal symptoms were observed through the use of validated outcome metrics across studies, which appears to be sustained. Additionally, no serious adverse events were reported in any of the studies. The most common adverse effect reported was post-procedural pain or discomfort. Further studies including randomized controlled studies are required in order to make a meta-analysis of the efficacy of ClariFix™ possible.

## Data Availability

All data was sourced from publicly available data.

## References

[1] Settipane RA (2009). Epidemiology of vasomotor rhinitis. World Allergy Organ J.

[2] Keith PK, Desrosiers M, Laister T, Schellenberg RR, Waserman S (2012). The burden of allergic rhinitis (AR) in Canada: perspectives of physicians and patients. Allergy Asthma Clin Immunol.

[3] Yan CH, Hwang PH (2018). Surgical management of nonallergic rhinitis. Otolaryngol Clin N Am.

[4] Lieberman P, Kaliner MA, Wheeler WJ (2005). Open-label evaluation of azelastine nasal spray in patients with seasonal allergic rhinitis and nonallergic vasomotor rhinitis. Curr Med Res Opin.

[5] Golding-Wood PH (1961). Observations on petrosal and vidian neurectomy in chronic vasomotor rhinitis. J Laryngol Otol.

[6] Kompelli AR, Janz TA, Rowan NR, Nguyen SA, Soler ZM (2018). Cryotherapy for the treatment of chronic rhinitis: a qualitative systematic review. Am J Rhinol Allergy.

[7] Terao A, Meshitsuka K, Suzaki H, Fukuda S (1983). Cryosurgery on postganglionic fibers (posterior nasal branches) of the pterygopalatine ganglion for vasomotor rhinitis. Acta Otolaryngol.

[8] Ow RA, O'Malley EM, Han JK, Lam KK, Yen DM (2021). Cryosurgical ablation for treatment of rhinitis: two-year results of a prospective multicenter study. Laryngoscope..

[9] Medical Devices Active Licence listing (MDALL)—CLARIFIX CRYOTHERAPY DEVICE [Internet]. Medical Devices Active Licence Listing (MDALL). Government of Canada [Cited 10 Dec 2022]. https://health-products.canada.ca/mdall-limh/.

[10] Gerka Stuyt JA, Luk L, Keschner D, Garg R (2021). Evaluation of in-office cryoablation of posterior nasal nerves for the treatment of rhinitis. Allergy Rhinol.

[11] Chang MT, Song S, Hwang PH (2020). Cryosurgical ablation for treatment of rhinitis: a prospective multicenter study. Laryngoscope.

[12] Singh AK, Kasle DA, Torabi SJ, Manes RP (2021). Adverse events associated with ClariFix posterior nasal nerve cryoablation: a MAUDE database analysis. Otolaryngol Head Neck Surg..

[13] Wilson KF, Spector ME, Orlandi RR (2011). Types of rhinitis. Otolaryngol Clin N Am.

[14] Del Signore AG, Greene JB, Russell JL, Yen DM, O'Malley EM, Schlosser RJ. Cryotherapy for treatment of chronic rhinitis: 3‐month outcomes of a randomized, sham‐controlled trial. In: Smith T, editor. International forum of allergy & rhinology, vol 12, no 1. Wiley; 2022. pp. 51–61. 10.1002/alr.22868 PMC9291981 34355872

[15] Hwang PH, Lin B, Weiss R, Atkins J, Johnson J. Cryosurgical posterior nasal tissue ablation for the treatment of rhinitis. In: Smith T, editor. International forum of allergy & rhinology, vol 7, no 10. Wiley; 2017. pp. 952–956. 10.1002/alr.21991 PMC5656830 28799727

[16] Yen DM, Conley DB, O’Malley EM, Byerly TA, Johnson J (2020). Multiple site cryoablation treatment of the posterior nasal nerve for treatment of chronic rhinitis: an observational feasibility study. Allergy Rhinol..

[17] Virani FR, Wilson MD, Beliveau AM, Gill AS, Strong EB, Steele TO. The impact of surgical posterior nasal nerve cryoablation on symptoms and disease-specific quality of life in patients with chronic rhinitis. Ear Nose Throat J. 2021. 10.1177/01455613211018576. 10.1177/01455613211018576 PMC8958794 34128402

[18] Yoo F, Kuan EC, Batra PS, Chan CK, Tajudeen BA, Craig JR. Predictors of rhinorrhea response after posterior nasal nerve cryoablation for chronic rhinitis. In: Smith T, editor. International forum of allergy & rhinology, vol 10, no 7. Wiley; 2020. pp. 913–919. 10.1002/alr.22574 32445248

[19] Higgins JPT, Altman DG, Sterne JAC. Chapter 8: Assessing risk of bias in included studies. In: Higgins JPT, Green S, editors. Cochrane handbook for systematic reviews of interventions Version 5.1.0. Updated March 2011. The Cochrane Collaboration; 2011. www.handbook.cochrane.org.

[20] Wells G, Shea B, O’Connell J, et al. The Newcastle–Ottawa Scale (NOS) for assessing the quality of nonrandomised studies in meta-analysis. http://www.ohri.ca/programs/clinical_epidemiology/oxfordasp. Accessed 12 Feb 2021.

[21] Kennedy JL, Hubbard MA, Huyett P, Patrie JT, Borish L, Payne SC (2013). Sino-nasal outcome test (SNOT-22): a predictor of postsurgical improvement in patients with chronic sinusitis. Ann Allergy Asthma Immunol.

[22] Gage AA, Baust JM, Baust JG (2009). Experimental cryosurgery investigations in vivo. Cryobiology.

[23] Leila R, Raluca P, De Greef Yves SD, Bruno S (2015). Cryoablation versus radiofrequency ablation in AVNRT: same goal, different strategy. J Atr Fibrillation..

[24] Byun H, Chung JH, Lee SH, Ryu J, Kim C, Shin JH (2021). Association of hypertension with the risk and severity of epistaxis. JAMA Otolaryngol Head Neck Surg.

[25] Lieberman P. Treatment update: nonallergic rhinitis. In: Allergy and asthma proceedings, vol, no 4. OceanSide Publications; 2001. p. 199. 11552668

[26] Su WF, Liu SC, Hsu WC, Chen YC (2014). Randomized, double-blind, controlled study to evaluate the effect of vidian nerve cauterization on lacrimation. Am J Rhinol Allergy.

[27] Tan G, Ma Y, Li H, Li W, Wang J (2012). Long-term results of bilateral endoscopic vidian neurectomy in the management of moderate to severe persistent allergic rhinitis. Arch Otolaryngol Head Neck Surg.

[28] Jang TY, Kim YH, Shin SH (2010). Long-term effectiveness and safety of endoscopic vidian neurectomy for the treatment of intractable rhinitis. Clin Exp Otorhinol.

[29] Faweett JW, Keynes RJ (1990). Peripheral nerve regeneration. Annu Rev Neurosci.

